# Effects of Age-Related Macular Degeneration on Postural Sway

**DOI:** 10.3389/fnhum.2017.00158

**Published:** 2017-03-31

**Authors:** Hortense Chatard, Laure Tepenier, Olivier Jankowski, Antoine Aussems, Alain Allieta, Talal Beydoun, Sawsen Salah, Maria P. Bucci

**Affiliations:** ^1^UMR 1141, Institut National de la Santé et de la Recherche Médicale—Université Paris 7, Robert Debré University HospitalParis, France; ^2^Vestibular and Oculomotor Evaluation Unit, ENT Department, Robert Debré University HospitalParis, France; ^3^Centre Ophtalmologique du Val-d'Oise (OPH95)Osny, France; ^4^Groupe Hospitalier Cochin-Hôtel-Dieu, Department of Ophthalmology, Assistance Publique-Hôpitaux de Paris, Paris Descartes UniversityParis, France

**Keywords:** age-related macular degeneration, postural sway, elderly, visual condition, balance

## Abstract

**Purpose:** To compare the impact of unilateral vs. bilateral age-related macular degeneration (AMD) on postural sway, and the influence of different visual conditions. The hypothesis of our study was that the impact of AMD will be different between unilateral and bilateral AMD subjects compared to age-matched healthy elderly.

**Methods:** Postural stability was measured with a platform (TechnoConcept®) in 10 elderly unilateral AMD subjects (mean age: 71.1 ± 4.6 years), 10 elderly bilateral AMD subjects (mean age: 70.8 ± 6.1 years), and 10 healthy age-matched control subjects (mean age: 69.8 ± 6.3 years). Four visual conditions were tested: both eyes viewing condition (BEV), dominant eye viewing (DEV), non-dominant eye viewing (NDEV), and eyes closed (EC). We analyzed the surface area, the length, the mean speed, the anteroposterior (AP), and mediolateral (ML) displacement of the center of pressure (CoP).

**Results:** Bilateral AMD subjects had a surface area (*p* < 0.05) and AP displacement of the CoP (*p* < 0.01) higher than healthy elderly. Unilateral AMD subjects had more AP displacement of the CoP (*p* < 0.05) than healthy elderly.

**Conclusions:** We suggest that ADM subjects could have poor postural adaptive mechanisms leading to increase their postural instability. Further studies will aim to improve knowledge on such issue and to develop reeducation techniques in these patients.

## Introduction

Age-Related Macular Degeneration (AMD) is the first cause of blindness after fifty years old in developed countries (Kocur and Resnikoff, 2002; Augood et al., 2006). This pathology is characterized by uni- or bi-lateral photoreceptor degeneration, which generates a large scotoma including central vision (Leveziel et al., 2009). Peripheral vision is conserved. AMD is a multifactorial and polygenic pathology with three main risk factors: age, environment and genetics (Chakravarthy et al., 2007; Wang et al., 2008, 2009). AMD represents a true public health issue because of the prevalence (1.6% before 64 years old and 27.9% after 85 years old; Ferris, 1983; Hyman and Neborsky, 2002; Friedman et al., 2004), the cost of care (which increases with disease severity; Bandello et al., 2008), psychological impact and functional disability (difficulty reading, driving restriction, difficulty of stereoscopic vision, difficulty recognizing faces, etc.; Augustin et al., 2007; Christoforidis et al., 2011; Hochberg et al., 2012; Sengupta et al., 2014; McCloud and Lake, 2015). This pathology affects more than one million people in France.

According to HAS (*Haute Autorité de Santé)* and other authors, 33% of subjects older than 65 years have experienced at least one fall per year (Tinetti et al., 1988; Campbell et al., 1989; Wood et al., 2011). It is a real public health problem because of autonomy loss and of the medical cost ($6–8 billion by year in the United States alone; Carroll et al., 2005).

Postural control is an elaborated process which allows a coordinated relationship of body segments (static and dynamic positions; Paillard, 1971; Gurfinkel and Shik, 1973). It is controlled by vestibular, proprioceptive, and visual information (Nashner, 1979; Horak and Shupert, 1994; Fetter and Dichgans, 1996). The vestibular system contributes to postural stability with eyes open (Fitzpatrick and McCloskey, 1994). Vision and proprioception participate to the detection of slow movements in the visual environment. When the visual or the vestibular system is affected, subjects need to compensate with other sensorial inputs (Brandt, 2003).

Some studies examined the impact of AMD on postural control (Elliott et al., 1995; Wood et al., 2009; Kotecha et al., 2013). Elliott et al. (1995) explored balance control (anterior-posterior sways of CoP) in AMD subjects compared to age-matched control subjects on a stable/unstable platform. They showed that postural stability in AMD subjects was poor when the inputs of kinesthetic sensory system were disrupted. The authors suggested that in normal standing condition, the vestibular and kinesthetic systems compensated for the lack of visual information in AMD subjects. Wood et al. (2009) studied postural stability in older adults with age-related maculopathy in order to identify the visual factors associated with postural control and falls. They proved that diminution of contrast sensitivity and visual field loss lead to postural instability and mobility difficulties. Kotecha et al. (2013) examined the effect of a secondary task on standing balance in elderly subjects with central visual field loss (AMD) or peripheral visual field loss (glaucoma) compared with age-matched healthy subjects. They compared two standing conditions: eyes open on a firm or a foam surface. These authors found that during the secondary task, AMD subjects were more unstable than healthy elderly on a firm and foam surface, while glaucoma subjects were more unstable on the foam surface only. Authors suggested that when subjects have visual impairment, they have to increase somatosensory contribution to obtain a good postural stability, and that peripheral vision is important when somatosensory inputs are disturbed.

The role of central vs. peripheral vision information in control of movements and posture was examined in numerous studies (i.e., Berencsi et al., 2005; Marigold and Patla, 2007). These authors suggested that peripheral vision is used for postural control and most particularly for stabilization of fore-aft sways; central vision is more used for foot trajectory planning, targeting, obstacle avoidance, and for stabilization of lateral sways.

Taken together all these findings showed poor postural stability in patients with AMD, particularly under eyes open condition; the novelty of the present study was to explore further AMD pathology (i) unilateral vs. bilateral AMD (ii) and the effect of different visual condition (both eyes open, and one eye alternatively open, dominant and non-dominant).

The hypothesis of our study was that the impact of AMD could be different between unilateral and bilateral AMD subjects compared with age-matched healthy elderly, and that postural sways could be different for different eye viewing conditions.

## Materials and methods

### Subjects

A total of 10 unilateral AMD patients between 62.8 and 76.7 years old (mean age: 71.1 ± 4.6 years) and 10 bilateral AMD patients between 57.1 and 78.5 years old (mean age: 70.8 ± 6.1 years) participated in the study. We also tested 10 age-matched healthy controls (mean age: 69.8 ± 6.3 years). All participants were recruited from the Department of Ophthalmology, Hôtel-Dieu Hospital in Paris and from the *Centre Ophtalmologique du Val-d'Oise* (France). Their participation was voluntary.

All participants had to fulfill criteria: ametropia inferior to five dioptries (spherical equivalent), no ocular surgery background, no retina laser treatment, no other ophthalmology pathologies, no diabetes, no known cognitive loss, no known vestibular abnormality, and no known orthopedic surgeries and abnormalities.

The investigation adhered to the principles of the Declaration of Helsinki and was approved by our Institutional Human Experimentation Committee (*Comité de Protection des Personnes CPP, Ile de France V*). Written informed consent was obtained from each participant after an explanation of the experimental procedure.

### Ophthalmologic and orthoptic evaluation

All AMD subjects underwent ophthalmologic and orthoptic examination to evaluate their visual function. Clinical data of each AMD patients are shown in Tables 1, 2. Clinical data of healthy elderly subjects are shown in Table 3.

**Table 1:** **Clinical characteristics of unilateral AMD subjects**.

**Patient (Age, years)**	**ETDRS**		**Glasses correction**		**AMD level**		**Type of AMD**	**Scotoma**		**Stereoacuity (TNO)**	**Eye dominant**
S1 (62.8)	RE:	20/40	RE:	+1.75 (−0.75) 100°	RE:	3	CNV	RE:	Perimacular	200″	LE
	LE:	20/20	LE:	+1.5 (−1.75) 85°	LE:	1	/	LE:	/		
S2 (63.8)	RE:	20/125	RE:	+2.25 (−0.25) 130°	RE:	4	GA	RE:	Perimacular	/	LE
	LE:	20/20	LE:	+2 (−0.5) 60°	LE:	1	/	LE:	/		
S3 (63.5)	RE:	20/20	RE:	+1.25 (−0.25) 80°	RE:	2	/	RE:	/	/	RE
	LE:	20/32	LE:	+1.5 (−0.25) 85°	LE:	4	CNV	LE:	/		
S4 (70.5)	RE:	20/40	RE:	+0.75 (−0.75) 40°	RE:	4	CNV	RE:	Perimacular	/	LE
	LE:	20/25	LE:	+2.5 (−0.75) 160°	LE:	2	/	LE:	/		
S5 (72.4)	RE:	20/20	RE:	−0.5 (−0.5) 105°	RE:	1	/	RE:	/	/	RE
	LE:	20/32	LE:	+0.5 (−0.75) 80°	LE:	4	CNV	LE:	Paramacular		
S6 (72.5)	RE:	20/25	RE:	+4.75	RE:	3	GA	RE:	/	480″	LE
	LE:	20/20	LE:	+4.75 (−0.75) 130°	LE:	2	/	LE:	/		
S7 (72.7)	RE:	20/20	RE:	+2.5 (−1) 90°	RE:	1	/	RE:	/	/	RE
	LE:	20/50	LE:	+2.75 (−1.5) 100°	LE:	4	CNV	LE:	Perimacular		
S8 (73.4)	RE:	20/40	RE:	+1.75 (−0.75) 80°	RE:	4	CNV	RE:	Perimacular	480″	LE
	LE:	20/40	LE:	+2 (−1)110°	LE:	3	/	LE:	/		
S9 (76.3)	RE:	20/32	RE:	+3 (−0.75) 95°	RE:	3	CNV	RE:	/	480″	LE
	LE:	20/20	LE:	+3.25 (−0.75) 125°	LE:	1	/	LE:	/		
S10 (76.7)	RE:	20/20	RE:	+0.75 (−0.75) 115°	RE:	1	/	RE:	/	480″	RE
	LE:	20/40	LE:	+0.75 (−0.75) 60°	LE:	4	CNV	LE:	Paramacular		

*Data are reported for each participant with unilateral AMD. Their corrected visual acuity (ETDRS), glasses correction, AMD level, type of AMD (GA, geographic atrophy; CNV, choroidal neovascularization), stereoscopic acuity (seconds of arc, TNO test) and dominant eye are reported (LE, left eye; RE, right eye)*.

**Table 2:** **Clinical characteristics of bilateral AMD subjects**.

**Patient (Age, years)**	**ETDRS**		**Glasses correction**		**AMD level**		**Type of AMD**	**Scotoma**		**Stereoacuity (TNO)**	**Dominant eye**
S11 (57.1)	RE:	20/40	RE:	+5 (−0.5) 80°	RE:	4	CNV	RE:	Perimacular	/	LE
	LE:	20/20	LE:	+3.5 (−0.5) 105°	LE:	3	CNV	LE:	Paramacular		
S12 (65.9)	RE:	20/800	RE:	+1.75 (−0.5) 110°	RE:	4	CNV	RE:	Perimacular	/	LE
	LE:	20/100	LE:	+1 (−0.75) 150°	LE:	4	CNV	LE:	Perimacular		
S13 (69.6)	RE:	20/20	RE:	+2 (−0.5) 130°	RE:	3	GA	RE:	Perimacular	480″	RE
	LE:	20/25	LE:	+2 (−0.5) 50°	LE:	3	GA	LE:	Perimacular		
S14 (69.8)	RE:	20/800	RE:	/	RE:	4	CNV	RE:	Paramacular	/	LE
	LE:	20/50	LE:	/	LE:	3	CNV	LE:	Perimacular		
S15 (69.9)	RE:	20/25	RE:	+0.5 (−0.75) 95°	RE:	3	CNV	RE:	/	480″	RE
	LE:	20/63	LE:	+0.75 (−0.5) 90°	LE:	4	CNV	LE:	/		
S16 (71.7)	RE:	20/800	RE:	/	RE:	4	GA	RE:	Para- and perimacular	/	RE
	LE:	20/320	LE:	/	LE:	4	GA	LE:	Paramacular		
S17 (74.1)	RE:	20/125	RE:	/	RE:	3	GA	RE:	Perimacular	/	RE
	LE:	20/20	LE:	+0.5 (−0.25) 145°	LE:	3	CNV	LE:	Paramacular		
S18 (74.8)	RE:	20/25	RE:	+1.25 (−0.75) 100°	RE:	3	GA	RE:	/	480″	RE
	LE:	20/32	LE:	+1.25 (-0.75) 70°	LE:	3	GA	LE:	Paramacular		
S19 (76.4)	RE:	20/800	RE:	/	RE:	4	CNV	RE:	Perimacular	/	LE
	LE:	20/32	LE:	/	LE:	3	GA	LE:	Paramacular		
S20 (78.5)	RE:	20/63	RE:	/	RE:	3	CNV	RE:	Perimacular	/	RE
	LE:	20/63	LE:	/	LE:	4	CNV	LE:	/		

*Data are reported for each participant with bilateral AMD. Their corrected visual acuity (ETDRS), glasses correction, AMD level, type of AMD (GA, geographic atrophy; CNV, choroidal neovascularization), stereoscopic acuity (seconds of arc, TNO test) and dominant eye are reported (LE, left eye; RE, right eye)*.

**Table 3:** **Clinical characteristics of age-matched healthy subjects**.

**Patient (Age, years)**	**ETDRS**		**Glasses correction**		**AMD level**		**Scotoma**		**Stereoacuity (TNO)**	**Dominant eye**
S21 (60.1)	RE:	20/20	RE:	(−0.75) 88°	RE:	1	RE:	/	480″	LE
	LE:	20/20	LE:	(−0.75) 100°	LE:	1	LE:	/		
S22 (63.2)	RE:	20/20	RE:	+1.50 (−0.25) 65°	RE:	1	RE:	/	480″	LE
	LE:	20/20	LE:	+2.50 (−1.5) 160°	LE:	1	LE:	/		
S23 (64.9)	RE:	20/20	RE:	+3.25 (−0.5) 105°	RE:	1	RE:	/	480″	RE
	LE:	20/20	LE:	+3.25 (−0.5) 80°	LE:	1	LE:	/		
S24 (66.5)	RE:	20/20	RE:	+0.50 (−0.5) 80°	RE:	1	RE:	/	480″	LE
	LE:	20/20	LE:	+0.25	LE:	1	LE:	/		
S25 (67.8)	RE:	20/20	RE:	+2.50 (−0.5) 60°	RE:	1	RE:	/	480″	RE
	LE:	20/20	LE:	+3 (−0.75) 105°	LE:	1	LE:	/		
S26 (69.8)	RE:	20/20	RE:	+0.25 (−0.5) 10°	RE:	1	RE:	/	480″	LE
	LE:	20/20	LE:	(−0.25) 150°	LE:	1	LE:	/		
S27 (69.9)	RE:	20/20	RE:	+1.25	RE:	1	RE:	/	480″	LE
	LE:	20/20	LE:	+1.25	LE:	1	LE:	/		
S28 (77.2)	RE:	20/20	RE:	+3	RE:	1	RE:	/	480″	RE
	LE:	20/20	LE:	+3	LE:	1	LE:	/		
S29 (79.2)	RE:	20/20	RE:	−1.5	RE:	1	RE:	/	480″	LE
	LE:	20/20	LE:	−1.75	LE:	1	LE:	/		
S30 (79.5)	RE:	20/20	RE:	+2.75 (−2) 5°	RE:	1	RE:	/	480″	RE
	LE:	20/20	LE:	+0.5 (−1.5) 20°	LE:	1	LE:	/		

*Data are reported for each healthy age-matched participant. Their corrected visual acuity (ETDRS), glasses correction, AMD level, stereoscopic acuity (seconds of arc, TNO test) and dominant eye are reported (LE, left eye; RE, right eye)*.

Visual acuity was measured separately for each eye at far distance (5 m) with the Monoyer chart. Next we have translated to ETDRS with an adapted scale. Stereoscopic acuity was measured by TNO test (Test of Netherlands Organization for Applied Scientific Research; Walraven, 1975). Unilateral AMD patients have a corrected monocular visual acuity between 20/125 and 20/20, and bilateral AMD patients a corrected monocular acuity between 20/800 and 20/25. Only eight of the ten AMD participants have a stereoscopic acuity <480 s of arc. Visual functions are also evaluated for control subjects. They have a monocular corrected visual acuity of 20/20 and stereoscopic acuity for 120 s of arc.

Age-related macular degeneration severity scale of AREDS was used for each eye (AREDS, 2001). SD-OCT (Spectralis®, Heidelberg Engineering) for each eye allows identifying AMD level by locating geographic atrophies (deterioration of the photoreceptors) and choroidal neovascularization (growth of pathologic blood vessels from the choroid into the subretinal space).

Among participants, 60% of bilateral AMD and 80% of unilateral AMD are choroidal neovascularization. Other studies have reported that there is two AMD with choroidal neovascularization for one AMD with geographic atrophy (Chakravarthy et al., 2007).

The eye with the better corrected visual acuity is considered as the dominant eye.

### Posturography

**Figure 1:**
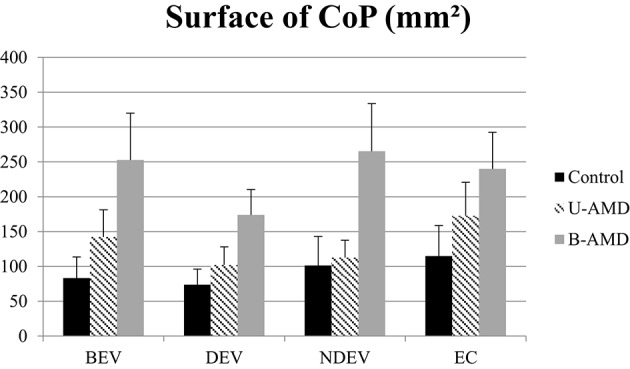
**Surface of CoP**. Mean value of the surface of CoP (mm^2^) for each group of subject tested (control age-matched elderly, unilateral AMD and bilateral AMD), for each visual conditions, binocular eye viewing (BEV), dominant eye viewing (DEV), non-dominant eye viewing (NDEV), eyes closed (EC). Vertical bars indicate the standard error.

A force platform (AFP40/16 Stabilotest, principle of strain gauge) consisting of two dynamometric clogs was used to measure and quantify postural stability (Standards by *Association Française de Posturologie*, produced by TechnoConcept®, Céreste, France; Figure 1). Foot position is standardized with footprints. This platform included a 16-bit analog-digital and acquisition frequency was 40 Hz. The excursion of center of pressure was measured during 25.6 s. Postural parameters were calculated following Gagey's standards (Gagey et al., 1993; Gagey and Weber, 1999).

### Postural recording procedure

In a dark room, participants stood on the platform and fixed a target (3 × 3 cm; identically for all subjects) in front of their eye level (150 cm). Four visual conditions were tested: binocular eye viewing (BEV), dominant eye viewing (DEV), non-dominant eye viewing (NDEV), and eyes closed (EC). We choose to test postural control separately for each eye in order to compare the impact of level of AMD on postural stability in order to develop training techniques for these subjects, even if these conditions are not physiological. Subjects were instructed to stay as still as possible with their arms along their body, to fix the target and stand quietly on the platform. Three randomized trials were performed for each visual condition successively. A short break was done between each condition. The total duration of the trial was 10 min.

### Data processing

To quantify the effect of AMD and visual conditions on postural control we analyzed the surface area (mm^2^), the length (mm), the mean speed (mm/s), and the anteroposterior (AP) and mediolateral (ML) displacements (mm) of the CoP that are the standard deviation of the displacement. Surface area is an effective measurement of CoP variability and corresponds to an ellipse with 90% of CoP (Chiari et al., 2002; Gagey and Weber, 2004; Vuillerme et al., 2008). Length is a path of CoP. Mean speed is an efficient indicator to quantity the neuro-muscular activity required to regulate postural control (Geurts et al., 1993).

### Statistical analysis

Data were analyzed with ANOVA/MANOVA using the three groups of subjects (unilateral, bilateral AMD subjects, and control subjects) as inter- subject factor, and the four visual conditions (both eyes opens, dominant and non-dominant eye open, and both eyes closed) as within-subject factor.

In the case of significant effects *post-hoc* Bonferroni test was performed. The effect of a factor was considered as significant when the *p*-value was below 0.05.

## Results

ANOVA test failed to show significant age differences between the three groups [*F*_(2, 27)_ = 0.66, *p* = 0.54].

Figure 1 shows the surface area of the CoP (mm^2^) for each visual condition tested (BEV, DEV, NDEV, EC) for the three groups of subjects (control, unilateral AMD, bilateral AMD). The analysis of variance (ANOVA) indicated a group effect [*F*_(2, 27)_ = 3.28, *p* < 0.05]. *Post-hoc* comparison showed a significant difference between “Control” and “Bilateral AMD” (*p* < 0.05): bilateral AMD subjects had a larger surface area than control subjects. There was a significant effect of visual condition [*F*_(3.81)_ = 3.04, *p* < 0.03]. *Post-hoc* comparison showed that surface area of CoP was significantly smaller under DEV with respect to EC (*p* < 0.02). ANOVA did not show any significant interaction between group and visual condition [*F*_(6.81)_ = 0.69, *p* = 0.65].

**Figure 2:**
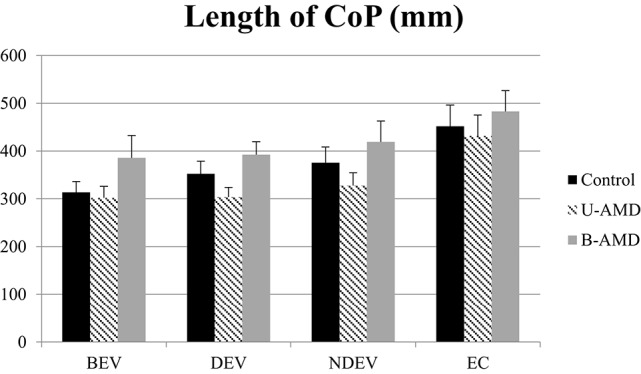
**Length of CoP**. Mean of the Length of CoP (mm^2^) for each group of subject tested (control age-matched elderly, unilateral AMD and bilateral AMD), for each visual conditions, binocular eye viewing (BEV), dominant eye viewing (DEV), non-dominant eye viewing (NDEV), eyes closed (EC). Vertical bars indicate the standard error.

Figure 2 shows the length of the CoP (mm) for each visual condition tested (BEV, DEV, NDEV, EC) for the three groups of subjects (control, unilateral AMD, bilateral AMD). ANOVA did not show a significant group effect [*F*_(3.81)_ = 2.29, *p* = 0.1] but indicated a significant effect of visual condition [*F*_(3.81)_ = 18.69, *p* < 10^−6^]. *Post-hoc* comparison showed that the length of the CoP was significantly smaller under BEV than under NDEV (*p* < 0.03) and under EC (*p* < 10^−6^). The length of the CoP was also significantly larger under EC than under DEV (*p* < 10^−6^) and NDEV (*p* < 10^−4^). ANOVA did not show a significant interaction between group and visual condition [*F*_(6.81)_ = 0.64, *p* = 0.67].

**Figure 3:**
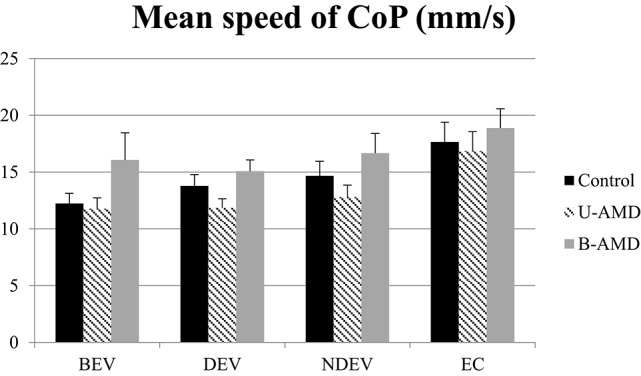
**Mean speed of CoP**. Mean of the mean speed of CoP (mm^2^) for each group of subject tested (control age-matched elderly, unilateral AMD and bilateral AMD), for each visual conditions, binocular eye viewing (BEV), dominant eye viewing (DEV), non-dominant eye viewing (NDEV), eyes closed (EC). Vertical bars indicate the standard error.

Figure 3 shows the mean speed of the CoP (mm/s) for each visual condition tested (BEV, DEV, NDEV, EC) in the three groups of subjects (control, unilateral AMD, bilateral AMD). The analysis of variance (ANOVA) did not show a significant group effect [*F*_(2, 27)_ = 2.88, *p* = 0.07] but indicated an effect of visual condition [*F*_(3, 81)_ = 9.68, *p* < 10^−4^]. *Post-hoc* comparison showed that the mean speed of the CoP was higher under EC than under BEV (*p* < 10^−4^), under DEV (*p* < 10^−4^), and under NDEV (*p* < 10^−2^). There was no significant interaction between group and visual condition [*F*_(6, 81)_ = 0.42, *p* = 0.85].

**Figure 4:**
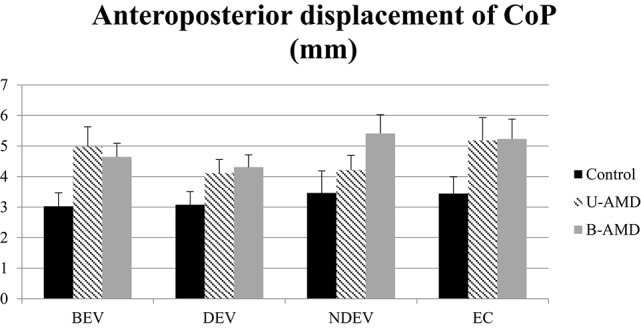
**AP displacements of CoP**. AP displacements of CoP (mm) for each group of subject tested (control age-matched elderly, unilateral AMD and bilateral AMD), for each visual conditions, binocular eye viewing (BEV), dominant eye viewing (DEV), non-dominant eye viewing (NDEV), eyes closed (EC). Vertical bars indicate the standard error.

Figure 4 shows the AP displacements of the CoP (mm) for each visual condition tested (BEV, DEV, NDEV, EC) in the three groups of subjects (control, unilateral AMD, bilateral AMD). The analysis of variance (ANOVA) indicated a significant group effect [*F*_(2, 27)_ = 3.43, *p* < 0.04]. The AP displacement was larger in AMD subjects than in healthy control age-matched subjects: *post-hoc* comparison showed that AP displacement of the CoP was shorter in control subjects than in bilateral AMD subjects (*p* < 0.01) and unilateral AMD subjects (*p* < 0.05). There was no significant effect of visual condition [*F*_(2, 27)_ = 2.51, *p* = 0.06] or interaction between group and visual condition [*F*_(6, 81)_ = 1.4, *p* = 0.22].

**Figure 5:**
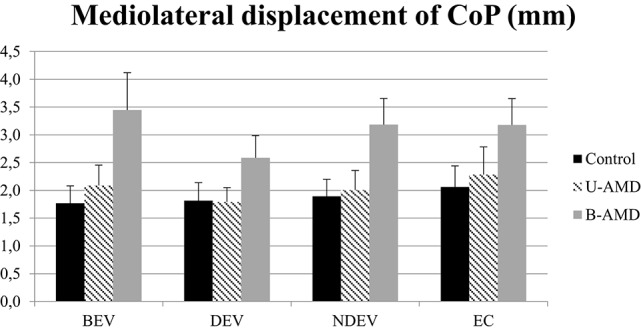
**ML displacements of CoP**. ML displacements of CoP (mm) for each group of subject tested (control age-matched elderly, unilateral AMD and bilateral AMD), for each visual conditions, binocular eye viewing (BEV), dominant eye viewing (DEV), non-dominant eye viewing (NDEV), eyes closed (EC). Vertical bars indicate the standard error.

Figure 5 shows the ML displacements of the CoP (mm) for each visual condition tested (BEV, DEV, NDEV, EC) in the three groups of subjects (control, unilateral AMD, bilateral AMD). The analysis of variance (ANOVA) did not show any significant group effect [*F*_(2, 27)_ = 2.64, *p* = 0.08], or any effect of visual condition [*F*_(3, 81)_ = 1.94, *p* = 0.1], or any interaction between group and visual condition [*F*_(6, 81)_ = 0.50, *p* = 0.8].

## Discussion

The main findings of this study are as follows: (i) the surface area and the AP displacement of the CoP are larger in bilateral AMD subjects than in unilateral AMD subjects; (ii) postural stability in elderly subjects depends on visual conditions. These findings are discussed individually below.

### Bilateral AMD subjects are more unstable than unilateral AMD subjects

In this study we found that AMD subjects had poor postural stability with respect to controls. This finding is in agreement with others studies (Elliott et al., 1995; Kotecha et al., 2013). Moreover, two postural parameters (surface area and AP displacement of CoP) were significantly different in bilateral AMD subjects compared with healthy elderly, and the AP displacement of CoP was significantly affected in unilateral AMD only.

Based on this finding we could assume that a postural evaluation, particularly of the surface area of the CoP, at the beginning of the AMD diagnosis may be predictive of future postural difficulties in these patients. An early postural rehabilitation care would prevent the risk of falling and in the future, studies leading with postural, and/or visual training will be necessary to improve the everyday life.

Postural balance changes throughout life. Qiu et al. (2012) studied the somatosensory system during aging and the impact of age on postural stability. Elderly patients (mean age: 72 years) have an augmentation of surface area and length of the CoP, and an augmentation of AP and ML sways, in comparison with young adults (mean age: 27 years). These authors suggested that mechanoreceptors sensibility decreases with aging as well as the capacity of treatment of sensorial information by the central nervous system. According to Faraldo-García et al. (2016), older subjects could have poor ability of adapt their body to disturbed sensory situations. Note that in younger subjects with loss of central vision (Stargardt disease) Agostini et al. (2016) found that compensatory strategies are used to control their postural stability. Such adaptive mechanisms are working well also in children with strabismus (see works of our groups, i.e., Lions et al., 2014; Bucci et al., 2016); we could make the hypothesis that in older subjects with AMD pathologies such compensations are not well developed, most likely because at this age plasticity occurs less frequently.

### Postural stability in elderly subjects depends on visual conditions

Our results proved that AP displacements of CoP was higher in AMD subjects than controls in closed eyes condition, most likely because AMD subjects have low mobility and degraded physical performance. Such hypothesis is confirmed by previous studies (Rovner et al., 2009; Chomistek et al., 2013; Loprinzi et al., 2015). These authors suggested that physical inactivity facilitated the progression of vision loss (Chomistek et al., 2013) and cognitive loss like depressive disorders (Rovner et al., 2009). Moreover, (Loprinzi et al., 2015) showed that AMD subjects, and more generally subjects with low visual acuity increased sedentary behavior, leading to increase of risk of developing metabolic, cardiovascular, and cerebrovascular diseases.

Few studies examined postural stability under monocular viewing. (Moraes et al., 2009), studied the impact of binocular vs. monocular viewing in controlling posture in quiet stance in young adults without visual abnormalities (mean age: 22.7 years). They suggested that binocular viewing allowed a greater postural control.

Note that even if our results failed to show any interaction effect between subjects and visual condition data on monocular viewing, they suggested that AMD subjects are more stable under dominant eye viewing than under both eyes viewing condition. Studies with more patients are needed to confirm this result. We made the hypothesis that monocular visual field of the dominant eye is less disturbed than binocular visual field in AMD subjects. The confirmation of this result would open perspectives of developing of training techniques without replacing the standard follow-up of the AMD subjects by clinical ophthalmological examination. In fact, in theory, neutralization process is expected to erase some of the scotoma (no view area) in the binocular visual field. But this process is difficult for elderly people due to the age-related decrease of brain plasticity.

The role of central vs. peripheral vision information in control balance was examined in several studies. Marigold and Patla (2007) examined the role of central or peripheral vision to avoid an obstacle. These authors reported that peripheral vision was sufficient for successful obstacle avoidance during locomotion. Moreover, more recently, Timmis et al. (2016) proved, in young adults, the impact of visual field loss (to 10° compared 20°) on risk of falls. They showed that only visual field loss to 20° increased risk of falls. We could hypothesis that the size of scotoma in AMD subjects may be predictive of postural instability. According to Berencsi et al. (2005), in young adults, central, and peripheral vision contributes to maintaining a stable standing posture. They suggested that peripheral vision control more the AP than ML displacements of CoP. Actually our result contrast this one, AP displacement of CoP is larger in AMD subjects with central field loss could be due to the different age of subjects tested in the two studies. Indeed, it is well-known that older subjects used more hip strategies to maintain postural control whereas young adults used ankle strategies (Daubney and Culham, 1999). Further studies comparing young and old subjects with poor vision are needed to explore postural strategies.

## Limitations

It is important to note that in this study we used a platform with a frequency of 40 Hz and this could explain the small displacement and mean speed values reported here comparing to others studies. Secondly, a larger number of subjects with AMD will be necessary to explore further their postural instability in relationship with their scotoma measures.

## Conclusion

The present study showed that AMD subjects, suffering from visual impairment in the central but not in the peripheral field, had worse postural performance than healthy age-matched control subjects, especially in the surface area (unilateral and bilateral AMD subjects) and AP displacements of the CoP (bilateral AMD subjects only). Because of aging, AMD subjects could have poor postural adaptive mechanisms which increase instability and risk of falls. Further studies will aim to improve knowledge and to develop reeducation techniques in these patients.

## Author contributions

HC, LT, OL, AnA, AlA, TB, SS, MB: substantial contributions to the conception of the word, logistic support for the recruitment of participants, acquisition, analysis and interpretation of data for the work, drafting the work and revising critically for important intellectual content, final approval of the version to be published, Agreement to be accountable for all aspects of the work in ensuring that questions related to the accuracy or integrity of any part of the work are appropriately investigated and resolved.

### Conflict of interest statement

The authors declare that the research was conducted in the absence of any commercial or financial relationships that could be construed as a potential conflict of interest.
